# Crystal structure of (4*Z*)-4-[(2*E*)-1-hydroxy-3-(naphthalen-2-yl)prop-2-en-1-yl­idene]-3-methyl-1-phenyl-1*H*-pyrazol-5(4*H*)-one

**DOI:** 10.1107/S205698901500866X

**Published:** 2015-05-09

**Authors:** Muhammad Salim, Munawar Ali Munawar, Muhammad Nawaz Tahir, Muhammad Shahid, Khizar Iqbal Malik

**Affiliations:** a Department of Chemistry, University of the Punjab, Lahore, Punjab, Pakistan; b Department of Physics, University of Sargodha, Sargodha, Punjab, Pakistan

**Keywords:** crystal structure, pyrazole, intra­molecular hydrogen bonding, π–π stacking

## Abstract

In the title compound, C_23_H_18_N_2_O_2_, the pyrazole ring subtends dihedral angles of 2.01 (13) and 1.55 (10)° with the pendant benzene ring and the naphthalene ring system, respectively. The mol­ecule is almost planar (r.m.s. deviation for the 27 non-H atoms = 0.025 Å) and intra­molecular O—H⋯O and C—H⋯O hydrogen bonds both close *S*(6) loops. In the crystal, very weak aromatic π–π stacking inter­actions between the benzene and the pyrazole rings, with centroid–centroid distances of 3.8913 (14) and 3.9285 (15) Å, are observed.

## Related literature   

For related structures, see: Chaudhry *et al.* (2012 ▸); Holzer *et al.* (1999 ▸); Malik *et al.* (2009 ▸).
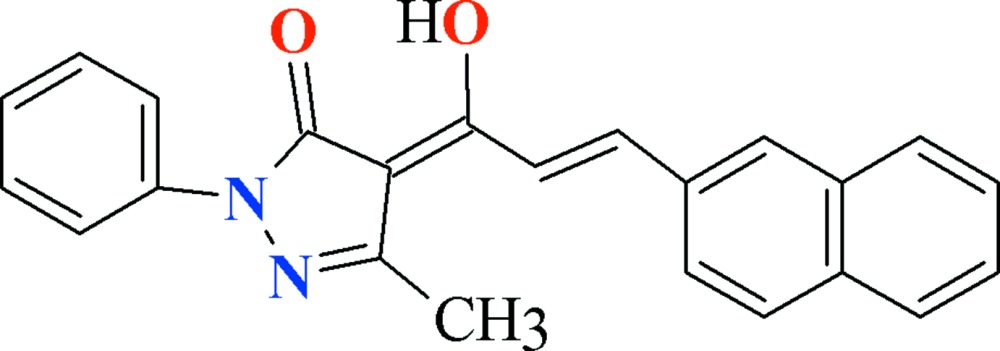


## Experimental   

### Crystal data  

C_23_H_18_N_2_O_2_
*M* *_r_* = 354.39
Monoclinic, 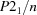

*a* = 6.7067 (8) Å
*b* = 17.525 (2) Å
*c* = 15.784 (2) Å
β = 101.152 (6)°
*V* = 1820.1 (4) Å^3^
*Z* = 4
Mo *K*α radiation
μ = 0.08 mm^−1^
*T* = 296 K
0.40 × 0.16 × 0.14 mm

### Data collection  

Bruker Kappa APEXII CCD diffractometer
Absorption correction: multi-scan (*SADABS*; Bruker, 2005 ▸) *T*_min_ = 0.968, *T*_max_ = 0.986
13979 measured reflections
3574 independent reflections
1855 reflections with *I* > 2σ(*I*)
*R*_int_ = 0.055

### Refinement  

*R*[*F* ^2^ > 2σ(*F* ^2^)] = 0.056
*wR*(*F* ^2^) = 0.140
*S* = 0.99
3574 reflections
246 parameters
H-atom parameters constrained
Δρ_max_ = 0.13 e Å^−3^
Δρ_min_ = −0.16 e Å^−3^

### 

Data collection: *APEX2* (Bruker, 2007 ▸); cell refinement: *SAINT* (Bruker, 2007 ▸); data reduction: *SAINT*; program(s) used to solve structure: *SHELXS97* (Sheldrick, 2008 ▸); program(s) used to refine structure: *SHELXL2014* (Sheldrick, 2015 ▸); molecular graphics: *ORTEP-3 for Windows* (Farrugia, 2012 ▸) and *PLATON* (Spek, 2009 ▸); software used to prepare material for publication: *WinGX* (Farrugia, 2012 ▸) and *PLATON*.

## Supplementary Material

Crystal structure: contains datablock(s) global, I. DOI: 10.1107/S205698901500866X/hb7418sup1.cif

Structure factors: contains datablock(s) I. DOI: 10.1107/S205698901500866X/hb7418Isup2.hkl

Supporting information file. DOI: 10.1107/S205698901500866X/hb7418Isup3.cml

**Fig. 1.**
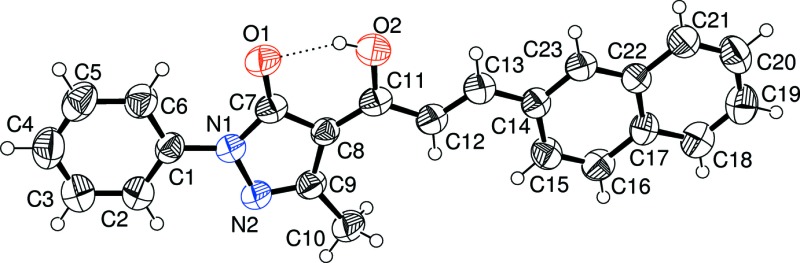
View of the title compound with displacement ellipsoids drawn at the 50% probability level.

. DOI: 10.1107/S205698901500866X/hb7418fig1.tif
View of the title compound with displacement ellipsoids drawn at the 50% probability level.

**Fig. 2.**
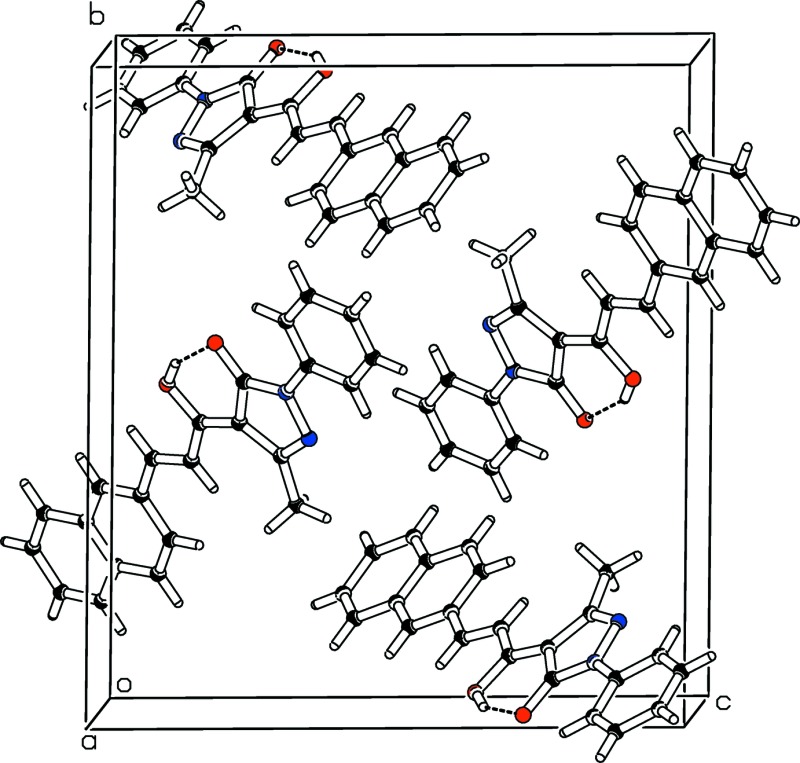
The partial packing, showing π–π interactions.

. DOI: 10.1107/S205698901500866X/hb7418fig2.tif
The partial packing, showing π–π inter­actions.

CCDC reference: 1062997

Additional supporting information:  crystallographic information; 3D view; checkCIF report

## Figures and Tables

**Table 1:** Hydrogen-bond geometry (, )

*D*H*A*	*D*H	H*A*	*D* *A*	*D*H*A*
O2H2*A*O1	0.82	1.80	2.555(2)	153
C6H6O1	0.93	2.30	2.940(3)	126

## supplementary crystallographic information

### S1. Comment

The crystal structures of 5-methyl-2-phenyl-4-((*E*)-3-phenyl-2-hydroxy- prop-2-enylidene)-1,2-dihydro-3*H*-pyrazol-3-one (Holzer *et al.*, 1999), (4*Z*)-4-((2*E*)-1-hydroxy-3-(4-methoxyphenyl)prop-2-en-1- ylidene)-3-methyl-1-phenyl-1*H*-pyrazol-5(4*H*)-one (Malik *et al.*, 2009) and (4*Z*)-4-((2*E*)-1-hydroxy-3-(3-nitrophenyl)prop- 2-en-1-ylidene)-3-methyl-1-(4-methylphenyl)-1*H*-pyrazol-5(4*H*)-one (Chaudhry, *et al.*, 2012) have been published which are related to the title compound (I, Fig. 1). (I) is synthesized for the biological studies as well as for the preparation of different metal complexes.

In (I), the benzene ring A (C1–C6) and the (4*Z*)-4-[(2*E*)-1- hydroxy-3-(naphthalen-2-yl)prop-2-en-1-ylidene]-5-methyl-2,4-dihydro-3*H* -pyrazol-3-one moiety *B* (C7 –C23/N1/N2/O1/O2) are almost planar with r.m.s. deviations of 0.0022 and 0.0179 Å, respectively. The dihedral angle between A/B is 2.30 (13)°. There exist intramolecular H-bonding of O—H···O type completing *S* (6) loop. There exist π–π interactions at a distance of 3.9285 (15) Å between the centroids of Cg1—Cg2^i^ and Cg2— Cg1^ii^ [i = 1 + x, y, z and ii = -1 + x, y, z], where Cg1 and Cg2 are the centroids of heterocyclic ring *C* (N1/N2/C7/C8/C9) and benzene ring *A* (Fig. 2). Similarly, there exist π–π interactions at a distance of 3.8913 (14) Å between the centroids of Cg3—Cg1^i^ and Cg1— Cg3^ii^ [i = 1 + x, y, z and ii = -1 + x, y, z], where Cg3 is the centroids of ring *D* (C14/C15/C16/C17/C22/C23).

### S2. Experimental

4-Acetyl-3-methyl-1-phenyl-5-hydroxy pyrazole (0.218 g, 1 mmol), 2-naphthaldehyde (0.234 g, 1.5 mmol) in glacial acetic acid (10 ml) and concentrated sulfuric acid (0.2 ml) was stirred at 353–360 K for 8 h. The reaction mixture was diluted with distilled water (50 ml). The precipitate was filtered, washed with methanol and dried. The crude product was purified by column chromatography using n-hexane and ethyl acetate mixtures as eluents. The product was recrystallized form n-hexane solution to afford purple needle. Yield = 56%, m.p. = 491 K

### S3. Refinement

The H-atoms were positioned geometrically (C–H = 0.93–0.96 Å, O—H= 0.82 Å) and refined as riding with *U*_iso_(H) = *xU*_eq_(C, O), where *x* = 1.5 for methyl and hydroxy and *x* =1.2 for other H-atoms.

### Crystal data

**Table:**

C_23_H_18_N_2_O_2_	*F*(000) = 744
*M**_r_* = 354.39	*D*_x_ = 1.293 Mg m^−^^3^
Monoclinic, *P*2_1_/*n*	Mo *K*α radiation, λ = 0.71073 Å
*a* = 6.7067 (8) Å	Cell parameters from 1855 reflections
*b* = 17.525 (2) Å	θ = 2.6–26.0°
*c* = 15.784 (2) Å	µ = 0.08 mm^−^^1^
β = 101.152 (6)°	*T* = 296 K
*V* = 1820.1 (4) Å^3^	Needle, purple
*Z* = 4	0.40 × 0.16 × 0.14 mm

### Data collection

**Table:**

Bruker Kappa APEXII CCD diffractometer	3574 independent reflections
Radiation source: fine-focus sealed tube	1855 reflections with *I* > 2σ(*I*)
Graphite monochromator	*R*_int_ = 0.055
Detector resolution: 7.80 pixels mm^-1^	θ_max_ = 26.0°, θ_min_ = 2.6°
ω scans	*h* = −8→5
Absorption correction: multi-scan (*SADABS*; Bruker, 2005)	*k* = −21→21
*T*_min_ = 0.968, *T*_max_ = 0.986	*l* = −19→19
13979 measured reflections	

### Refinement

**Table:**

Refinement on *F*^2^	Primary atom site location: structure-invariant direct methods
Least-squares matrix: full	Secondary atom site location: difference Fourier map
*R*[*F*^2^ > 2σ(*F*^2^)] = 0.056	Hydrogen site location: inferred from neighbouring sites
*wR*(*F*^2^) = 0.140	H-atom parameters constrained
*S* = 0.99	*w* = 1/[σ^2^(*F*_o_^2^) + (0.0552*P*)^2^] where *P* = (*F*_o_^2^ + 2*F*_c_^2^)/3
3574 reflections	(Δ/σ)_max_ < 0.001
246 parameters	Δρ_max_ = 0.13 e Å^−^^3^
0 restraints	Δρ_min_ = −0.16 e Å^−^^3^

### Special details

**Table:**

Geometry. All e.s.d.'s (except the e.s.d. in the dihedral angle between two l.s. planes) are estimated using the full covariance matrix. The cell e.s.d.'s are taken into account individually in the estimation of e.s.d.'s in distances, angles and torsion angles; correlations between e.s.d.'s in cell parameters are only used when they are defined by crystal symmetry. An approximate (isotropic) treatment of cell e.s.d.'s is used for estimating e.s.d.'s involving l.s. planes.
Refinement. Refinement of *F*^2^ against ALL reflections. The weighted *R*-factor *wR* and goodness of fit *S* are based on *F*^2^, conventional *R*-factors *R* are based on *F*, with *F* set to zero for negative *F*^2^. The threshold expression of *F*^2^ > σ(*F*^2^) is used only for calculating *R*-factors(gt) *etc*. and is not relevant to the choice of reflections for refinement. *R*-factors based on *F*^2^ are statistically about twice as large as those based on *F*, and *R*-factors based on ALL data will be even larger.

### Fractional atomic coordinates and isotropic or equivalent isotropic displacement parameters (Å^2^)

**Table:**

	*x*	*y*	*z*	*U*_iso_*/*U*_eq_	
O1	0.1673 (2)	0.54343 (9)	0.17724 (11)	0.0684 (5)	
O2	0.4599 (3)	0.49546 (9)	0.10912 (11)	0.0672 (5)	
H2A	0.3651	0.5215	0.1186	0.101*	
N1	0.1223 (3)	0.46669 (10)	0.29460 (12)	0.0527 (5)	
N2	0.2155 (3)	0.40098 (11)	0.33743 (12)	0.0593 (6)	
C1	−0.0446 (3)	0.49941 (13)	0.32414 (15)	0.0510 (6)	
C2	−0.1094 (4)	0.46827 (15)	0.39423 (17)	0.0714 (8)	
H2	−0.0430	0.4260	0.4221	0.086*	
C3	−0.2734 (4)	0.49985 (18)	0.42332 (19)	0.0851 (9)	
H3	−0.3152	0.4788	0.4711	0.102*	
C4	−0.3740 (4)	0.56112 (17)	0.3831 (2)	0.0800 (8)	
H4	−0.4843	0.5818	0.4028	0.096*	
C5	−0.3108 (4)	0.59203 (15)	0.3130 (2)	0.0764 (8)	
H5	−0.3794	0.6339	0.2851	0.092*	
C6	−0.1459 (4)	0.56188 (14)	0.28280 (16)	0.0639 (7)	
H6	−0.1040	0.5835	0.2353	0.077*	
C7	0.2133 (3)	0.48753 (12)	0.22764 (15)	0.0508 (6)	
C8	0.3725 (3)	0.43280 (12)	0.22720 (14)	0.0462 (6)	
C9	0.3620 (3)	0.38203 (12)	0.29714 (15)	0.0533 (6)	
C10	0.4899 (4)	0.31336 (14)	0.32827 (16)	0.0743 (8)	
H10A	0.4788	0.2767	0.2824	0.111*	
H10B	0.4434	0.2910	0.3764	0.111*	
H10C	0.6293	0.3286	0.3458	0.111*	
C11	0.4960 (3)	0.43911 (13)	0.16670 (15)	0.0503 (6)	
C12	0.6639 (3)	0.38941 (12)	0.15888 (15)	0.0535 (6)	
H12	0.6932	0.3489	0.1974	0.064*	
C13	0.7796 (3)	0.39858 (13)	0.09910 (15)	0.0532 (6)	
H13	0.7481	0.4397	0.0617	0.064*	
C14	0.9479 (3)	0.35123 (12)	0.08677 (15)	0.0490 (6)	
C15	1.0124 (4)	0.28705 (13)	0.13931 (16)	0.0590 (7)	
H15	0.9461	0.2750	0.1841	0.071*	
C16	1.1694 (4)	0.24273 (13)	0.12549 (17)	0.0607 (7)	
H16	1.2071	0.2005	0.1606	0.073*	
C17	1.2762 (3)	0.25934 (13)	0.05903 (16)	0.0519 (6)	
C18	1.4400 (4)	0.21453 (14)	0.04322 (18)	0.0667 (7)	
H18	1.4792	0.1716	0.0771	0.080*	
C19	1.5422 (4)	0.23311 (16)	−0.02099 (19)	0.0730 (8)	
H19	1.6492	0.2028	−0.0308	0.088*	
C20	1.4857 (4)	0.29768 (17)	−0.07192 (18)	0.0735 (8)	
H20	1.5571	0.3104	−0.1149	0.088*	
C21	1.3268 (4)	0.34231 (15)	−0.05928 (16)	0.0632 (7)	
H21	1.2905	0.3849	−0.0939	0.076*	
C22	1.2170 (3)	0.32398 (13)	0.00630 (14)	0.0491 (6)	
C23	1.0518 (3)	0.36872 (13)	0.02178 (14)	0.0518 (6)	
H23	1.0121	0.4111	−0.0128	0.062*	

### Atomic displacement parameters (Å^2^)

**Table:**

	*U*^11^	*U*^22^	*U*^33^	*U*^12^	*U*^13^	*U*^23^
O1	0.0733 (12)	0.0593 (11)	0.0730 (12)	0.0127 (9)	0.0152 (9)	0.0219 (9)
O2	0.0714 (14)	0.0630 (12)	0.0694 (12)	0.0042 (9)	0.0190 (9)	0.0172 (10)
N1	0.0513 (13)	0.0557 (12)	0.0505 (12)	0.0075 (10)	0.0082 (10)	0.0075 (10)
N2	0.0575 (14)	0.0632 (13)	0.0577 (13)	0.0130 (11)	0.0125 (10)	0.0150 (11)
C1	0.0440 (16)	0.0540 (15)	0.0523 (15)	0.0045 (12)	0.0028 (11)	−0.0076 (12)
C2	0.066 (2)	0.0815 (19)	0.0692 (19)	0.0154 (16)	0.0189 (15)	0.0112 (16)
C3	0.079 (2)	0.103 (2)	0.079 (2)	0.0145 (19)	0.0293 (17)	0.0075 (18)
C4	0.067 (2)	0.086 (2)	0.089 (2)	0.0127 (18)	0.0177 (17)	−0.0186 (19)
C5	0.073 (2)	0.0635 (18)	0.088 (2)	0.0187 (15)	0.0044 (16)	−0.0115 (17)
C6	0.0651 (18)	0.0612 (16)	0.0645 (17)	0.0099 (14)	0.0105 (13)	−0.0034 (14)
C7	0.0488 (16)	0.0475 (14)	0.0535 (15)	−0.0038 (12)	0.0029 (12)	0.0040 (12)
C8	0.0389 (14)	0.0490 (14)	0.0491 (14)	−0.0021 (12)	0.0051 (11)	0.0045 (11)
C9	0.0494 (16)	0.0521 (14)	0.0568 (15)	0.0034 (12)	0.0065 (12)	0.0079 (13)
C10	0.0706 (19)	0.0756 (18)	0.0787 (19)	0.0235 (15)	0.0194 (14)	0.0298 (15)
C11	0.0480 (16)	0.0451 (14)	0.0533 (15)	−0.0085 (12)	−0.0013 (12)	0.0003 (12)
C12	0.0501 (16)	0.0504 (14)	0.0584 (16)	−0.0051 (13)	0.0064 (12)	0.0034 (12)
C13	0.0521 (16)	0.0483 (14)	0.0581 (16)	−0.0096 (12)	0.0081 (12)	0.0002 (12)
C14	0.0445 (15)	0.0464 (14)	0.0552 (15)	−0.0089 (12)	0.0076 (12)	−0.0017 (12)
C15	0.0578 (17)	0.0536 (15)	0.0682 (17)	−0.0082 (13)	0.0185 (13)	0.0080 (13)
C16	0.0576 (17)	0.0504 (15)	0.0726 (18)	−0.0062 (14)	0.0088 (13)	0.0127 (13)
C17	0.0431 (15)	0.0480 (14)	0.0632 (17)	−0.0093 (12)	0.0070 (12)	−0.0082 (12)
C18	0.0623 (19)	0.0541 (16)	0.082 (2)	−0.0060 (14)	0.0104 (15)	−0.0042 (14)
C19	0.0643 (19)	0.0697 (19)	0.087 (2)	−0.0030 (15)	0.0187 (16)	−0.0200 (17)
C20	0.070 (2)	0.089 (2)	0.0660 (19)	−0.0130 (17)	0.0261 (15)	−0.0147 (17)
C21	0.0609 (18)	0.0693 (17)	0.0593 (17)	−0.0065 (15)	0.0113 (13)	−0.0028 (14)
C22	0.0466 (16)	0.0519 (15)	0.0475 (14)	−0.0113 (13)	0.0057 (11)	−0.0056 (12)
C23	0.0496 (16)	0.0482 (14)	0.0554 (16)	−0.0064 (12)	0.0042 (12)	0.0039 (12)

### Geometric parameters (Å, º)

**Table:**

O1—C7	1.262 (2)	C10—H10C	0.9600
O2—C11	1.332 (2)	C11—C12	1.447 (3)
O2—H2A	0.8200	C12—C13	1.342 (3)
N1—C7	1.368 (3)	C12—H12	0.9300
N1—C1	1.415 (3)	C13—C14	1.444 (3)
N1—N2	1.418 (2)	C13—H13	0.9300
N2—C9	1.312 (3)	C14—C23	1.381 (3)
C1—C2	1.377 (3)	C14—C15	1.414 (3)
C1—C6	1.383 (3)	C15—C16	1.360 (3)
C2—C3	1.387 (3)	C15—H15	0.9300
C2—H2	0.9300	C16—C17	1.410 (3)
C3—C4	1.359 (4)	C16—H16	0.9300
C3—H3	0.9300	C17—C18	1.412 (3)
C4—C5	1.371 (4)	C17—C22	1.416 (3)
C4—H4	0.9300	C18—C19	1.368 (3)
C5—C6	1.390 (3)	C18—H18	0.9300
C5—H5	0.9300	C19—C20	1.397 (3)
C6—H6	0.9300	C19—H19	0.9300
C7—C8	1.436 (3)	C20—C21	1.368 (3)
C8—C11	1.385 (3)	C20—H20	0.9300
C8—C9	1.430 (3)	C21—C22	1.418 (3)
C9—C10	1.504 (3)	C21—H21	0.9300
C10—H10A	0.9600	C22—C23	1.417 (3)
C10—H10B	0.9600	C23—H23	0.9300
			
C11—O2—H2A	109.5	O2—C11—C12	115.5 (2)
C7—N1—C1	130.2 (2)	C8—C11—C12	126.1 (2)
C7—N1—N2	111.32 (18)	C13—C12—C11	123.6 (2)
C1—N1—N2	118.43 (19)	C13—C12—H12	118.2
C9—N2—N1	106.08 (18)	C11—C12—H12	118.2
C2—C1—C6	119.4 (2)	C12—C13—C14	126.8 (2)
C2—C1—N1	119.8 (2)	C12—C13—H13	116.6
C6—C1—N1	120.8 (2)	C14—C13—H13	116.6
C1—C2—C3	120.1 (3)	C23—C14—C15	118.1 (2)
C1—C2—H2	119.9	C23—C14—C13	119.5 (2)
C3—C2—H2	119.9	C15—C14—C13	122.4 (2)
C4—C3—C2	121.0 (3)	C16—C15—C14	121.2 (2)
C4—C3—H3	119.5	C16—C15—H15	119.4
C2—C3—H3	119.5	C14—C15—H15	119.4
C3—C4—C5	119.1 (3)	C15—C16—C17	121.5 (2)
C3—C4—H4	120.4	C15—C16—H16	119.3
C5—C4—H4	120.4	C17—C16—H16	119.3
C4—C5—C6	121.1 (3)	C16—C17—C18	122.8 (2)
C4—C5—H5	119.4	C16—C17—C22	118.5 (2)
C6—C5—H5	119.4	C18—C17—C22	118.7 (2)
C1—C6—C5	119.3 (3)	C19—C18—C17	121.1 (3)
C1—C6—H6	120.3	C19—C18—H18	119.4
C5—C6—H6	120.3	C17—C18—H18	119.4
O1—C7—N1	127.1 (2)	C18—C19—C20	120.0 (3)
O1—C7—C8	127.3 (2)	C18—C19—H19	120.0
N1—C7—C8	105.57 (19)	C20—C19—H19	120.0
C11—C8—C9	135.0 (2)	C21—C20—C19	120.8 (3)
C11—C8—C7	119.6 (2)	C21—C20—H20	119.6
C9—C8—C7	105.3 (2)	C19—C20—H20	119.6
N2—C9—C8	111.71 (19)	C20—C21—C22	120.4 (2)
N2—C9—C10	118.5 (2)	C20—C21—H21	119.8
C8—C9—C10	129.8 (2)	C22—C21—H21	119.8
C9—C10—H10A	109.5	C17—C22—C23	118.8 (2)
C9—C10—H10B	109.5	C17—C22—C21	118.9 (2)
H10A—C10—H10B	109.5	C23—C22—C21	122.3 (2)
C9—C10—H10C	109.5	C14—C23—C22	121.9 (2)
H10A—C10—H10C	109.5	C14—C23—H23	119.0
H10B—C10—H10C	109.5	C22—C23—H23	119.0
O2—C11—C8	118.4 (2)		
			
C7—N1—N2—C9	0.1 (2)	C7—C8—C11—O2	−1.4 (3)
C1—N1—N2—C9	179.18 (19)	C9—C8—C11—C12	0.3 (4)
C7—N1—C1—C2	−179.6 (2)	C7—C8—C11—C12	178.99 (19)
N2—N1—C1—C2	1.5 (3)	O2—C11—C12—C13	0.8 (3)
C7—N1—C1—C6	1.2 (4)	C8—C11—C12—C13	−179.6 (2)
N2—N1—C1—C6	−177.66 (19)	C11—C12—C13—C14	−179.44 (19)
C6—C1—C2—C3	−0.6 (4)	C12—C13—C14—C23	−180.0 (2)
N1—C1—C2—C3	−179.7 (2)	C12—C13—C14—C15	−0.2 (4)
C1—C2—C3—C4	0.7 (4)	C23—C14—C15—C16	−1.3 (3)
C2—C3—C4—C5	−0.3 (4)	C13—C14—C15—C16	179.0 (2)
C3—C4—C5—C6	−0.2 (4)	C14—C15—C16—C17	0.8 (4)
C2—C1—C6—C5	0.1 (3)	C15—C16—C17—C18	179.8 (2)
N1—C1—C6—C5	179.3 (2)	C15—C16—C17—C22	0.2 (3)
C4—C5—C6—C1	0.3 (4)	C16—C17—C18—C19	−178.9 (2)
C1—N1—C7—O1	1.4 (4)	C22—C17—C18—C19	0.7 (3)
N2—N1—C7—O1	−179.63 (19)	C17—C18—C19—C20	0.4 (4)
C1—N1—C7—C8	−178.9 (2)	C18—C19—C20—C21	−1.0 (4)
N2—N1—C7—C8	0.0 (2)	C19—C20—C21—C22	0.4 (4)
O1—C7—C8—C11	0.5 (3)	C16—C17—C22—C23	−0.7 (3)
N1—C7—C8—C11	−179.13 (19)	C18—C17—C22—C23	179.63 (19)
O1—C7—C8—C9	179.5 (2)	C16—C17—C22—C21	178.4 (2)
N1—C7—C8—C9	−0.1 (2)	C18—C17—C22—C21	−1.3 (3)
N1—N2—C9—C8	−0.2 (2)	C20—C21—C22—C17	0.7 (3)
N1—N2—C9—C10	−179.94 (18)	C20—C21—C22—C23	179.8 (2)
C11—C8—C9—N2	179.0 (2)	C15—C14—C23—C22	0.7 (3)
C7—C8—C9—N2	0.2 (3)	C13—C14—C23—C22	−179.51 (18)
C11—C8—C9—C10	−1.3 (4)	C17—C22—C23—C14	0.2 (3)
C7—C8—C9—C10	179.9 (2)	C21—C22—C23—C14	−178.8 (2)
C9—C8—C11—O2	179.9 (2)		

### Hydrogen-bond geometry (Å, º)

**Table:**

*D*—H···*A*	*D*—H	H···*A*	*D*···*A*	*D*—H···*A*
O2—H2*A*···O1	0.82	1.80	2.555 (2)	153
C6—H6···O1	0.93	2.30	2.940 (3)	126
