# Supplementary figures and images for: Crystal structure of (4Z)-4-[(2E)-1-hydroxy-3-(naphthalen-2-yl)prop-2-en-1-yl­idene]-3-methyl-1-phenyl-1H-pyrazol-5(4H)-one
Source: Acta Crystallogr E Crystallogr Commun. 2015 May 9;71(Pt 6):o381. doi: 10.1107/S205698901500866X (PMC4459333; doi:10.1107/S205698901500866X)

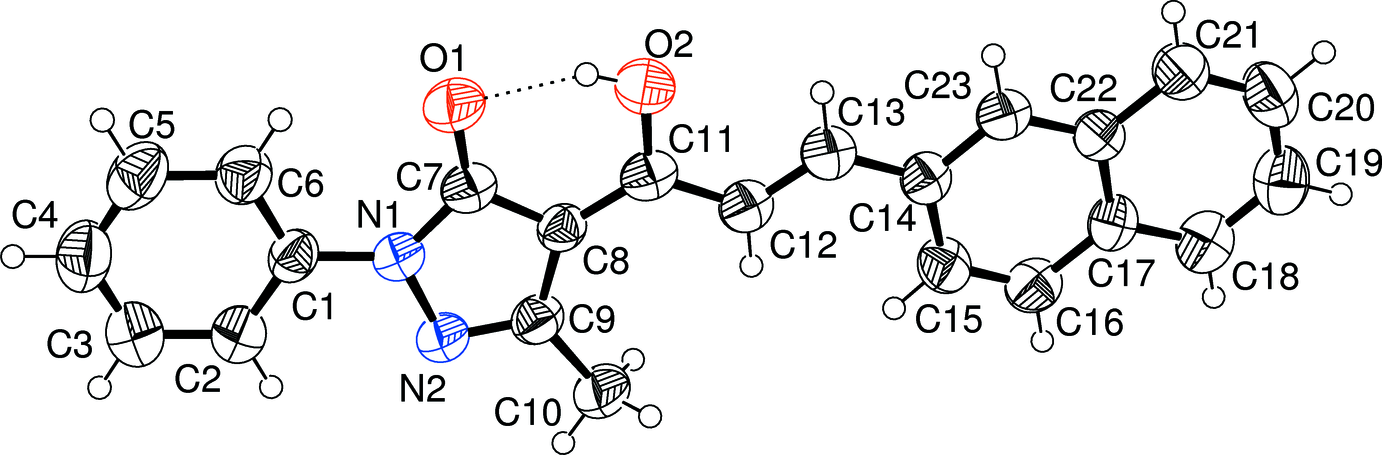

Supplement: Supplementary file 4 [file e-71-0o381-fig1.tif]

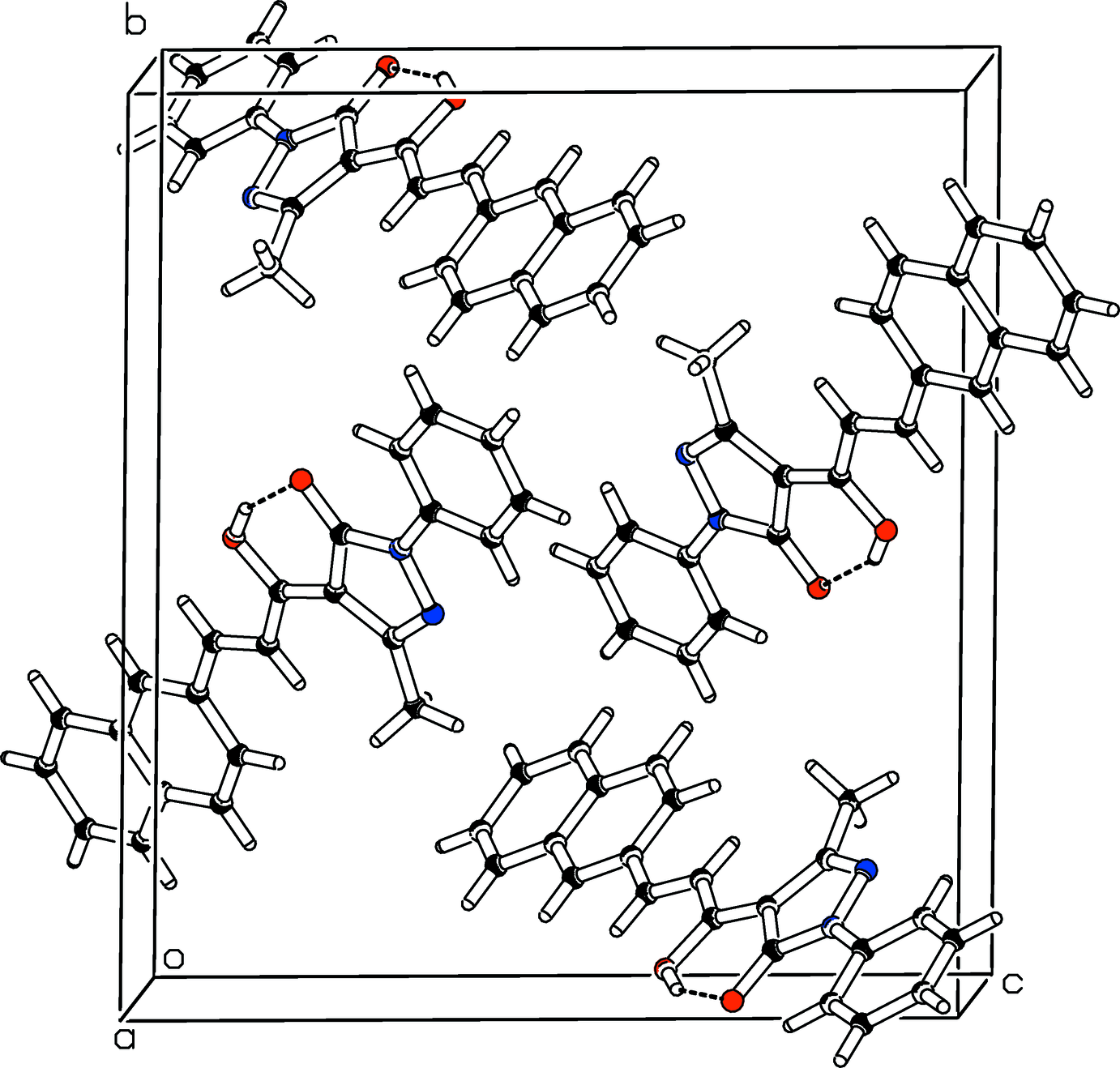

Supplement: Supplementary file 5 [file e-71-0o381-fig2.tif]
